# Exploring effectiveness of different health financing mechanisms in Nigeria; what needs to change and how can it happen?

**DOI:** 10.1186/s12913-019-4512-4

**Published:** 2019-09-13

**Authors:** Obinna Onwujekwe, Nkoli Ezumah, Chinyere Mbachu, Felix Obi, Hyacinth Ichoku, Benjamin Uzochukwu, Hong Wang

**Affiliations:** 1 Health Policy Research Group, Department of Pharmacology and Therapeutics, College of Medicine, University of Nigeria Enugu-Campus, Enugu, Nigeria; 2 Department of Community Medicine, College of Medicine, University of Nigeria Enugu-Campus, Enugu, Nigeria; 3 R4D, Abuja, Nigeria; 4 Department of Economics, University of Nigeria Nsukka, Nsukka, Nigeria; 5 Bill and Melinda Gates Foundation, Seattle, WA USA

**Keywords:** Health financing mechanisms, Performance, Needed change, Financial risk protection, Universal health coverage, Health system actors, Nigeria

## Abstract

**Background:**

Various attempts to examine health financing mechanisms in Nigeria highlight the fact that there is no single mechanism that fits all contexts and people. This paper sets out findings of an in-depth assessment of different health financing mechanisms in Nigeria.

**Methods:**

The study was undertaken in the Federal Capital territory of Nigeria and two States (Niger and Kaduna). Data were collected through review of government documents, and in-depth interviews of purposively selected respondents. Data analysis was guided by a conceptual framework which draws from various approaches for assessing health financing mechanisms. Data was examined for current practices, what needs to change and how the change can happen.

**Results:**

Health financing mechanisms in Nigeria do not operate optimally. Allocation and use of resources are neither evidence-based nor results-driven. Resources are not allocated equitably or in a manner that minimizes wastage and improves efficiency. None of the mechanisms effectively protects individuals/households from catastrophic health expenditure. Issues with social health insurance cut across legal frameworks and use of Health Maintenance Organisations (HMOs) as purchasers. The concomitant effect is that attainment of Universal Health Coverage is greatly compromised. In order to improve efficiency of health financing mechanisms, government needs to allocate more funds for purchasing health services; this spending must be based on evidence (strategic), and appropriately tracked. The legislation that established National Health Insurance Scheme should be amended such that social health insurance becomes mandatory for all citizens. Implementation of the latter should be complemented by revision of benefit package, strict oversight and regulation of HMOs.

**Conclusion:**

In order to improve health financing in the country, legal and regulatory frameworks need to be revised. Efficient utilization of resources could be improved through strategic purchasing arrangements and strict oversight.

## Background

It is important to understand the current status of health financing in Nigeria especially in comparison to national, regional and global goals and targets so as to provide evidence-based guidance for developing interventions that will be used to improve the financing of healthcare services in the country. In addition, since health financing is one of the building blocks of health system, its level of functionality has direct effect on the overall functioning of the health system. A persistent and major weakness of the country’s health system is the poor functioning of the health financing building block, which is characterized by low public spending, very high levels of out-pocket spending (one of the highest in the world), high incidence of catastrophic health spending and impoverishment due to spending on healthcare [1–4].

A missing gap in knowledge in Nigeria is a holistic understanding of political, economic and other institutional factors that either undermine or support the implementation of different financing mechanisms in different contexts in Nigeria. This knowledge will be invaluable in appropriately shaping Nigeria’s policy and programmatic choices that can improve health financing and accelerate the achievement of Universal Health Coverage (UHC) in the country. However, opportunities abound for Nigeria to increase coverage with social health insurance and other financial risk protection mechanisms and ultimately substantially improve the functioning of the health system with healthy citizens [3].

The political, economic and social contexts of health financing mechanisms are important considerations for selecting which mechanisms have the potential to succeed and survive [5, 6]. The manner in which health financing mechanisms are organized could affect other social goals and hence individual choices and options [7]. The two main sources of health financing in Nigeria, which are tax-based revenues and private contributions (from employers and individuals), lie closer to the country’s budgetary outcome on a causal chain that traces back to underlying social and political determinants [8]. The balance of these two sources, and the levels of funding available, are ultimately determined by political structures, institutions, power groups, legal commitments, and fiscal space [8]. Intentional review of these contextual factors will enable better understanding of conditions that enable or constrain sustainability of particular health financing mechanisms [9].

Efforts to increase public funding of health at all levels of government in Nigeria are being explored alongside other options for health financing such as public-private partnerships and overseas development assistance [10, 11]. Health financing mechanisms that aim at promoting equity are particularly likely to challenge vested interests of different actors [12, 13]. Actors’ views about what is desirable and feasible within contexts, and what is politically acceptable need to be taken into consideration in selecting appropriate health financing mechanisms [10, 11, 14–16]. It is necessary to identify potential actors, understand their relative power to influence change, their vested interests in the process and how this is related to the political and economic contexts in which they operate [12]. This will ensure that legitimate concerns and interests are considered, and alliances of support, sufficient to overcome potential opposition to change, are created [17–20].

The Nigerian National Health Insurance Scheme (NHIS) was established in 1999, and officially launched in 2005, to provide financial risk protection for citizens and reduce the high burden of out-of-pocket spending (OOPs) on individuals and households. In order to ensure that no one is left out, the NHIS has a number of programs namely, social health insurance for formal sector employees, community-based health insurance, private health insurance, and voluntary health insurance [16]. The NHIS’ objective of ensuring access to quality health services for all Nigerians has also been viewed as a positive step towards achieving universal health coverage (UHC) [9, 12, 21]. However, evidence shows that NHIS has been unable to achieve intended population coverage with financial risk protection [22]. Out-of-pocket expenditures constitute nearly 90% of the total private health spending, placing a significant burden on households, and about 60% of all health spending is financed directly by households without insurance [10, 11]. Ichoku and colleagues highlight that uneven distribution of resources in Nigeria has had an effect on health care financing, particularly OOPs [23].

Nigeria operates a three-tier federal system of government, and each tier is autonomous with executive and legislative arms. However, over the years, local governments, which constitute the third tier of government, have considerably lost their autonomy as successive state governments are having more control over local government administration and funding [24]. The Nigeria constitution places health on the concurrent legislative list, such that both the federal, state and local governments have the responsibility to mobilize and deploy resources for the provision of health services within their respective jurisdiction.

Understanding the effectiveness of different health financing mechanisms in Nigeria and the modifications in their functions that are needed to ensure improvements in financial risk protection are important. It is also useful to understand the bottlenecks that constrain the implementation of financing mechanisms such as social health insurance that can ensure financial risk protection to most Nigerians. These will help to significantly increase the level of financial risk protection in Nigeria in line with the requirement for achieving UHC.

This paper presents new information on the political, social and institutional contexts for health financing in Nigeria, which are essential evidence for improving financial risk protection for most citizens towards achievement of Universal Health Coverage in Nigeria. The paper also examines the specific characteristics of major health financing mechanisms in terms of mobilization, pooling of funds and purchasing functions. It also presents evidence on the performance of these health financing mechanisms as well as the political, economic and institutional issues constraining or facilitating implementation. It highlights actors and their roles in different health financing mechanisms, changes that need to occur with each mechanism and how these changes can happen to ensure financial risk protection for all citizens, towards achievement of universal health coverage in Nigeria.

## Methods

This study used a qualitative approach to examine the current situation of major health financing mechanisms in Nigeria, and future prospects for achieving universal financial risk protection for all citizens. Review of relevant national and state documents was complemented with in-depth interviews (IDI) of key informants. The use of multiple data collection methods provided complementarity, triangulation and validation of data. Whereas review of government documents provided data that represent program intent, key informant interviews supplied data that reflect actors’ interpretation and experience program implementation and performance. The study was undertaken in the Federal Capital Territory and two other states -Niger and Kaduna.

### Conceptual framework

The framework for conceptualization is drawn from Ribot’s programme paper on local actors, power and accountability [21], Leichter’s and Collins et al.’s models for categorizing contextual factors, WHO Health Financing Diagnostics and Guidance [21–24], and The World Bank’s core protocol for assessing performance of countries’ health financing systems [25]. Health care financing mechanisms and reforms evolve in many different contexts, and understanding the initial conditions in which they are being implemented or would be implemented is a useful starting point for assessing the reasons for pursuing them, the likely implications for the shape and pace of the mechanisms, and their potential sustainability in similar or different contexts. The nature of the system in which health financing mechanisms occur also affects their transitioning; while the institutional legacies shape the environment in which they unfold.

The Drivers of Change approach developed by DFID stipulates that thinking more systematically about how change occurs, the power relationships at stake and understanding institutional and structural factors affecting lack of political will leads to more realistic country assessments and planning, improved risk analysis, better prioritization, more realistic timeframes and the development of better-informed strategies to influence and support health reform programmes [26]. Hence, a better understanding of the context (including actors) in which health financing mechanisms operate in Nigeria will enable identification and development of acceptable and sustainable health financing model(s) towards quality and equitable health service delivery.

### Data collection

Data were collected through review of government documents and in-depth interviews of key informants.

#### Document review process

Detailed review of existing government documents was done to identify the current health financing mechanisms in Nigeria and their contributions to the overall funding for health, as well as all actors involved in health financing in Nigeria. A document review template was used to guide the review process.

#### In-depth interviews with key informants

The document review was followed by in-depth interviews of key informants to explore the roles of key actors in health financing and the influence of contextual factors in determining the performance of these mechanisms in terms of health systems goals of equity, efficiency, quality and sustainability. The in-depth interviews also explored perceptions of key stakeholders on future prospects of major health financing mechanisms for achieving universal financial risk protection in Nigeria.

A topic guide was developed for this study and used for in-depth interviews (Additional file 1). The guide was developed in English language and pretested to ensure clarity of questions and constructs. All interviews were conducted in English and audio-recorded with the consent of respondents to ensure that no relevant information was missed while taking hand-written notes.

Respondents were purposively selected based on their knowledge, experience, interest and active involvement in health financing in Nigeria. Key informants were drawn from different categories of health system actors to ensure maximum variability and representation of diverse perspectives. The respondents were public and private sector decision makers, particularly people in government, the labour unions and professional associations. Respondents were recruited from health facilities, health maintenance organizations (HMOs) and community groups. The Federal Ministry of Health (FMOH), the NHIS, the NPHCDA, the FCT health departments and State Ministries of Health were the major loci for data collection. Federal and State Ministries of Finance and Budget & Planning, and legislators from State and National Assembly were also included. A total of 32 respondents were interviewed.

### Data analysis

Qualitative data were transcribed and coded (manually and using NVivo). Audio-recorded interviews were transcribed verbatim and accuracy checks done to ensure their validity. A careful study of all the transcripts was undertaken in order to obtain a general view and make sense of the data. Transcripts that were particularly rich in information were studied in detail and responses coded. The codes were categorised and linked into clusters of relatively similar responses (themes and sub-themes) that represent key issues of institutional assessment of different financing mechanisms. These themes were used for coding and analysis of subsequent transcripts. The analysis of each major financing mechanism was undertaken in terms of health financing functions of resource generation/revenue mobilization; pooling and management of resources; and purchasing of services. Furthermore, the performance of each major health financing mechanism was analyzed in terms of equity, efficiency, sustainability, quality of services and ability to prevent consumers from incurring catastrophic health expenditures.

## Results

### Major health financing mechanisms in Nigeria

The major health financing mechanisms in Nigeria are namely: (i) government budget using general tax revenue; (ii) direct out-of-pocket payments; (iii) a social insurance scheme known as the Formal Sector Social Health Insurance Programme (FSSHIP) that is implemented by the National health insurance scheme; and (iv) donor funding. Other health financing mechanisms include: demand-side financing through conditional cash transfers (CCT), and community-based health insurance (CBHI). A summary of the characteristics of the health financing mechanisms with respect to health financing functions of resource generation, pooling and management of funds, and purchasing of health services is presented in Table 1.

**Table 1:** Characteristics of major health financing mechanisms in Nigeria

	Resource generation	Pooling and management of funds	Purchasing of health services
Government budget	Share of statutory allocation from FAAC Internally generated revenue from income tax, value-added tax, tariffs, sale of government bonds, etc. Special intervention funds	Funds are pooled into the federation account from where budgetary allocations are made. Disbursements are made quarterly from Ministry of Finance to Ministry of Health through the Central Bank	Health services are purchased through the Ministry of Health and related agencies for line items and global budget.
Out of pocket payment	Individual and households generate funds for health through: (i) income from paid employment and business, (ii) borrowing from family and friends, (iii) charity and philanthropy	Pooled within the household and managed by the head of the household or a representative. There is no central pool for revenue generated for OOP.	Individuals and households make direct cash payments at the point of accessing health care. Some health services like investigations and drugs could be provided through a third party such as diagnostic laboratory, pharmacy shop or patent medicine vendor.
FSSHIP Formally launched in 2005 Covers only Federal government employees and beneficiaries	Designed to be contributory – 10% from employers and 5% from employee of basic salary. However, only the employer is currently contributing (i.e. 10%).	Pooling is done centrally by NHIS into a dedicated Bank account	Health Maintenance Organizations (HMOs) are contracted to purchase health services (within an approved package of care) from accredited providers
Community-based health insurance	Funds are generated through contribution of premiums by registered enrolees.	Each scheme has its own pooling mechanism.	Depends on the design, but purchasing can be done directly from service providers or through third parties such as HMOs.
Donor funding	UN agencies through UNDP’s NEX Bilateral agencies - Country tax revenue Development Banks – contributions of member countries Other sources – philanthropists, donor cooperation, etc.	Each donor agency pools its fund separately and channels it through grants and concessional loans using aid modalities such as technical assistance, project financing, and little or no direct budget support. Development aid that is sent through regions to respective countries are managed by designated parastatals, specifically Ministry of Budget and National Planning in Nigeria.	Services are purchased through different models depending on financial risk assessment of recipient parastatal/organization. Direct implementation (by donors) or reimbursement models are used if financial risk is high, while direct transfers are used when financial risk is low

### Actors involved in health financing at the federal level

There are several actors within and external to the health sector that were identified as key role players in financing of the health sector in Nigeria. Actors within the health sector include the Federal Ministry of Health and its Agencies at all levels, Private Sector, Civil Society, Development Partners, and Academia. Actors external to the health sector include the Federal and State Governments, National Assembly, Ministry of Finance, Ministry of Budget and National Planning, Federal Inland Revenue Service, Customs, the Budget Office, Central Bank, Accountant General Office, Auditor General Office, among others. The key actors and their roles are summarized in Table 2.

**Table 2:** Roles of key actors in health financing in Nigeria

Actor category	Key players	Roles
Federal government and line Ministries	Federal Executive Council (FEC)	Approves policies that have macroeconomic and financial implications before operationalization
	Ministry of Finance	Critical role in advising FEC to ensure that health financing reforms align well with macroeconomic realities of the Country
	National Assembly (NASS)	Responsible for appropriation of budget to health sector and monitoring its implementation through standing (Senate or House) Committees on Health
Federal Ministry of Health and its Agencies	Federal Ministry of Health (FMOH)	Pivotal in health financing. Statutorily responsible for developing health policies and designing health programs and interventions. The health financing unit of FMOH is strategically positioned to promote the use of evidence in design and implementation of health financing reforms. It coordinates the Technical Working Group on health financing and engages other stakeholders to ensure support of health financing reforms. FMOH has been working with NHIS and NPHCDA in development of guidelines for management of Basic Healthcare Provision Fund (BHCPF).
	National Health Insurance Scheme (NHIS)	Runs and manages the Formal Sector Social Health Insurance Program (FSSHIP) and statutorily oversees the operations of HMOS in Nigeria. NHIS Zonal and State offices are positioned to provide needed support for State health insurance schemes.
	National Primary Health Care Development Agency (NPHCDA)	Focuses on improving quality and uptake of essential health services for vulnerable groups through interventions that incorporate both supply and demand-side financing such as the Midwives Service Scheme (MSS), Subsidy Reinvestment Program (SURE-P), and the Nigeria State Health Investment Project (NSHIP). To underscore the role of NPHCDA, a respondent said that *‘NPHCDA’s role is to ensure that services are provided at the PHC level. They have run performance-based financing pilots in some States, and the lessons learnt and experiences gained would be useful going forward”*
Development partners and other donor agencies		Involved in pooling and management of financial resources Technical expertise and support in health financing and public finance management. Technical support with strategic purchasing of services based on their experiences in using implementing partners (IPs) to deliver critical health interventions to Nigerians. Through this, they have been able to strengthen service delivery, contracting, logistics and payment mechanisms. *“For example, CHAI has helped to drive down the cost of drugs by creating a market for drugs. Reference pricing around products and drugs can help us do strategic purchasing of vaccines and essential drugs. DPs have gained experience with contracts with vendors and communities, and making use of their IPs, we can strike a lasting relationship in purchasing services which can help improve quality of health infrastructure and services”*
Private sector	Upstream actors (e.g. Private Sector Health Alliance)	The upstream actors are those involved in resource mobilization and domestic revenue mobilization, as well as investors. The upstream players also include foundations, and corporate organizations who earmark resources for corporate social responsibility activities.
	Downstream actors	The downstream players are mainly the service providers and it was acknowledged by some respondents that *“over 60–65% of health services are delivered by the private sector in Nigeria”*.
Health Maintenance Organizations		Interface between government and private providers of healthcare in the social health insurance schemes
Academia		Expand knowledge base and generate evidence to bridge the policy-research gap. Build capacity for health financing Serve as a repository of knowledge
Citizens and citizen groups	Civil Society Organizations (CSOs)	Ensure quality of care by guaranteeing accountability and value for money Informing and mobilizing citizens
	Media	Informing and mobilizing citizens
States and Local governments		Important roles in initiating and sustaining health financing reforms. *Who did you make policy for when all federal constituencies are situated within a state and their LGAs?”* States are expected to own and domesticate all health policies that are approved and adopted by the National Council on Health, to ensure proper implementation. CBHI and mutual aid are often managed at the LGA level by LGA health authority.

### Performance of major health financing mechanisms in Nigeria

A set of five key performance indicators were used to examine participants’ perspective of the major health financing mechanisms in Nigeria, and the findings are presented in detail below. The performance indicators that were used are efficiency, equity, quality of care, effect on catastrophic health expenditure, and sustainability. Table 3 shows a summary of perceived performance of each health financing mechanism.

**Table 3:** Participants’ perceptions of performance of major health financing mechanisms in Nigeria

	Government budget	OOP	FSSHIP	CBHI	Donor funding
Efficiency	Highly inefficient. Funds are inadequate and disproportionately allocated b/w personnel and service delivery. Allocation is not based on epidemiologic or demographic evidence. Hence, no value for money.	Regressive and highly inefficient. Pool is fragmented. Running cost is high.	NHIS has made efficiency gains but FSSHIP is inefficient because employees have not started contributing. Use of HMOs ↑s admin cost and ↓s what is available for service delivery. Difficult to ascertain value for money.	Efficiency is low because size of pool is too small	Opinions varied. Efficient because it employs cost-saving mechanisms to achieve high impact, and fiduciary and accountability requirements are strict. Inefficient because resources are sometimes wasted due to weak coordination of donor funds.
Equity	Inequitable. Mainly funds tertiary hospitals at the expense of needed primary care. No fairness in geographic distribution of resources.	Inequitable. Access to healthcare is determined by ability to pay.	Limited to FG employees and beneficiaries. Majority of Nigerians are not covered	Inequitable. Coverage is low	Donor funds are earmarked for specific services that do not benefit everyone.
Quality of care	Generally suboptimal but varies across facilities – quality of care is better in tertiary hospitals	Directly linked to affordability and availability of services	Benefit package is not comprehensive and quality of care is suboptimal	Depends on the scheme and process of implementation	Perceived to be relatively high.
Effect on household health expenditure	Has not reduced OOP or catastrophic health expenditure	High tendency for catastrophic health expenditure	Reduces direct OOP for enrollees for services covered	Risk protection for basic health services	Tendency to reduce direct OOP for services covered
Sustainability	As predictability (in time and amount) of funds. Perceived to be predictable in time but unpredictable in amount due to economic and political contexts	Not sustainable. Depends on ability of users to pay for health services	Current practice (FG statutory transfers) is unsustainable. HMOs are paid based on enrollees allocated rather than productivity	Not sustainable without cross-subsidization	Not sustainable. Lack of or delay in payment of counterpart funds by some State governments. Apparent donor fatigue

#### Government budget

##### Efficiency

The use of government budget for healthcare financing was perceived to be largely inefficient because disproportionately large proportion of the health budget is allocated to personnel cost without commensurate productivity, and the amount of money that is effectively available for service delivery is inadequate. Hence, value for money is difficult to ascertain and almost impossible to achieve under the current arrangement.

“*The health sector has one of the highest budgets for paying staff salaries compared to other ministries. We put so much into the health sector and we keep getting incessant strikes by doctors and other health workers*” (Government official).

Additionally, budget allocations to the health sector are not based on evidence of demographic considerations or epidemiological factors such as disease burden. As a result of this, government funding for health is grossly inadequate. According to a government official;

*“Sometimes it* (government budget for health) *is 4% of the total budget, sometimes, it is 6%. I think the best we ever had is about 7%”*.

##### Equity

National health budget was considered inequitable for a couple of reasons. Firstly, it mainly funds tertiary hospitals, whereas the highest disease burdens are attended to at the primary and secondary care levels. And although National Primary Health Care Development Agency (NPHCDA) gets a fraction of the annual health budget, the percentage of total health expenditure allocated to primary health care (PHC) was perceived to be incommensurate with disease burden.

“*Even though the small allocation to healt*h *sector, we think about 90% of Nigerians suffer from PHC challenges compared to tertiary challenges”*.

Secondly, it was stated that there is no system to ensure financial resources are fairly distributed across geographic regions in the country considering their differential poverty and vulnerability levels. This was clearly stated by a respondent;

*“There is inequity in the distribution of government’s resources and poverty level varies in different parts of the country. Some states are generating more money than others, and budget more for health. Some states are disadvantaged by geography and insecurity like those in the NE. We’re yet to finance healthcare in a way that ensures equity”* (Government official).

##### Quality of service

Although health services were perceived to be better in tertiary hospitals compared to primary and secondary hospitals, respondents inferred that quality of care varied from facility to facility depending on the dimension of quality assessed. Generally, they were of the opinion that quality of health care financed from government budget was sub-optimal in all public hospitals in terms of infrastructure, availability of drugs and skilled health workforce, waiting times and attitude of health workers towards clients.

##### Prevention of households from incurring catastrophic OOPs

The funding for health that is derived from government budget was perceived, in principle, to have no impact on OOP. This is because majority of the services provided in tertiary hospitals, for instance, are paid for through fee-for-service arrangements by individuals and households. For the very few public health interventions that are provided free (such as vaccination, family planning, HIV and TB treatment) service users are sometimes constrained to pay for ancillary services like laboratory tests, drugs and consumables.

##### Sustainability

This was expressed as predictability of government budget in time and amount dimensions. Whereas most respondents alluded to the fact that government budget is predictable in time due to legislative backing of government’s fiscal budget, predictability in terms of amount was said to be subject to trade-offs and easily influenced by economic situations and political interests of key decision makers such as legislators and bureaucrats in health and finance parastatals.

Consequently, a respondent suggested that; “*Health can be sustainably financed by increasing the fiscal space for health and expanding the revenue streams earmarked for health. There’s need to discuss at inter-sectoral level and get their buy-in and make an investment case for health in terms of its contribution to growth in GDP* (gross domestic product)”.

#### Formal sector social health insurance program (FSSHIP)

##### Efficiency

Although NHIS has made efficiency gains of several billions of Naira from the FSSHIP pool over the years, the scheme was considered inefficient. This is because currently, only the employer (federal government) is making contributions. Enrollees (employees) have not started making their contributions. On a different note, because unspent fee-for-service payments for beneficiaries could be statutorily retained by HMOs, they are faced with a disincentive to approve referrals from primary to secondary level care when needed by enrollees.

##### Equity

Majority of the respondents were of the opinion that although NHIS is designed such that benefit package is same for all enrolees, coverage of FSSHIP is only limited to Federal government employees and their dependents, and this category of people make up less than 5% of the population of Nigeria.

##### Quality of services

The perception of poor quality of services provided by NHIS-FSSHIP resonated among majority of respondents. With respect to drugs, it was reported that the NHIS formulary is not comprehensive, and essential drugs on the list were often out-of-stock in public hospitals. Hence, insured clients sometimes had to make direct cash payments for drugs. A government official stated that,

“The essential drugs are often out of stock in (public) health facilities and people are compelled to buy drugs (that are) not on the NHIS list”.

Long waiting times in public hospitals was also reported; and commenting on this, a government official said:

*“I walked into FMC Lokoja* (Kogi State) *and I saw over a hundred people waiting for treatment under the NHIS”.*

The poor quality of care was attributed to poor regulation of HMOs and providers by NHIS. This lack of regulation, in their opinion, is the reason why *“the HMOs are not addressing the delays* (in service delivery) *and quality of care received by beneficiaries”.* HMOs’ apathy towards improving service quality was perceived to be enabled by failure of clients to lodge complaints of dissatisfaction through established channels.

*“We have dedicated telephone lines but majority of enrollees don’t make complaints. Enrollees are afraid to make complaints and HMOs have capitalized on the lack of complaints”* (Government official)*.*

Clients’ lack of demand for better quality of care was linked to the fact that they have not started contributing their share of the premium.

*“They* (clients) *think it is* (FSSHIP) *a favour. No enrollee has contributed a dime, so people don’t think they are losing anything, hence not complaining or demanding for quality care”* (CSO)

Centralization of contractual arrangements with HMOs was perceived as an enabler of monopoly which makes HMOs too powerful to be effectively regulated by NHIS. According to a CSO official,

*“The HMOs have become so powerful that making progress on social health insurance has become difficult, and they will fight* (against) *it. HMOs are owned by powerful people who have influence in government”*.

##### Prevention of households from incurring catastrophic OOPs

Majority of respondents were of the opinion that the FSSHIP has been helpful in preventing enrollees from incurring out of pocket payments. Some of their thoughts are:

“*It has significantly reduced damaging out-of-pocket expenditure for beneficiaries. You only pay 10% co-insurance for drugs, some surgeries and for admissions. The first 15 days are free”* (Service user)*.*

“*I used XXX Hospital, and I paid only ₦120 for tests and drugs, but I still had to buy some drugs outside. If I was to pay for the tests and other drugs, it would have cost me ₦7000 outside, but how can a poor family without insurance pay?”* (Service user)

##### Sustainability

Although dependence on contributions from the federal government makes funding for the scheme predictable in principle, its long-term sustainability is highly amenable to fluctuations in government fiscal space. Additionally, the lack of contribution by enrollees inadvertently reduces the pool of funds and any prospects for sustainability, particularly now that NHIS’ expenses have exceeded revenue from premium contributions and reserved funds are being used to buffer the gap.

Overall, it appears that *“if money from the government stops, the scheme will collapse”*.

#### Donor funding

Development assistance for health (DAH) through bilateral and multilateral agencies and foundations is one of the major sources of funding for public health financing and population-based interventions in Nigeria.

##### Efficiency

There were varied opinions about the efficiency of donor funding. Some said that it is efficient and has saved government money, especially in the procurement of family planning commodities through special inexpensive price-reduction mechanisms. On the other hand, there were perceptions that donor funds were managed through multiple sources which are not coordinated, and government was lacking in showing leadership and coordination.

*“The support for health is insufficient and uncoordinated by government, and we have so many donors and CSOs like UNICEF, Save the Children, World Bank, DFID etc. who provide support … The government needs to provide leadership, ownership and coordination”* (CSO).

The failure of government to coordinate donor funding was attributed to challenges in its capacity to undertake this role.

*“Many times we don’t have capacity to monitor as money for the monitoring is not in the budget … It is very difficult to control donors. You can’t control he who feeds you*” (Government official).

##### Equity

Donor funds were perceived to be inequitable because funded activities are not implemented in all States and support is for specific services that may not benefit everyone. However, for the health services that are supported, some respondents were of the opinion that equity is achieved for targeted subpopulation groups.

##### Quality of health services

This was perceived to be high for the reason that health products that are of good standard are procured direct from manufacturers, and donors provide technical assistance for implementation through training and supportive supervision of health workers, as well as provision of service delivery guidelines. Donor funds were actually considered as filling a gap in service delivery, and as captured in this quote,

*“... With the advent of TB, Malaria and HIV/AIDS, they took over the management of the health sector and filled the vacuum with technical assistance and funding*”.

The challenge, however, is that donors sometimes do not align with National priorities and their good intentions may disrupt, rather than strengthen the health system. As indicated by a government official,

*“They* (donors) *are doing a lot but we are not managing it well, which gives them the leeway to do their own things. The NSHDP* (National Strategic Health Development Plan) *was to help get them into alignment with national plans but FMOH seems a bit too cowed and intimidated by donors. Donors have good intentions but disruptive if not well coordinated”.*

##### Prevention of households from incurring catastrophic OOP

To some extent donor funding was perceived to be responsive to the needs of people in the communities.

*“Development partners (DPs) have got some grassroots engagement and are deeply rooted in the communities than the government and understand the communities. People respond to them because DPs brought what they needed”* (CSO)*.*

For this reason, donor funding was considered to contribute in reducing damaging OOP for target subpopulation groups.

##### Sustainability

Sustainability of donor funded programs was a major concern for most respondents because of the level of dependence of the Nigerian health system on donor funding which was reported to be dwindling. According to a government official,

*“The donors are tired and not ready to give money again to Nigeria. It is embarrassing that FMOH depends on donors a lot, and Nigeria is not fulfilling her own obligations*”.

Although there are mechanisms, such as counterpart funding and basket of funds, which have been introduced to ensure sustainability of development aid, compliance from State governments has been poor. Some State governments do not contribute their share of counterpart funds, and some others make their contribution later than agreed.

The Delivery as One (DAO) strategy which was introduced by UN is geared towards ensuring that all UN agencies have one voice, one office, one strategic plan and one basket of funds. The ‘one basket of funds’ component of the strategy is yet to be implemented.

#### Out of pocket payment (OOPS)

##### Efficiency

It was considered by all respondents as one of the most inefficient ways of financing healthcare, because the pool is fragmented with no efficiency gains due to high running cost on the part of healthcare providers.

##### Equity

OOP worsens existing inequities in access to available health services because ability to pay determines access to healthcare. Hence, the richest have better access (in terms of range and quality) to healthcare than the poorest.

##### Quality of service

This was perceived to vary from facility to facility. Health providers/facilities that charge higher user fees were considered more likely to procure services of higher quality than those that charge lower user fees. As a development partner respondent said

“*People simply buy healthcare from wherever they find it from any provider for an acceptable quality”.*

##### Prevention of households from incurring catastrophic OOP

It was perceived to lead to impoverishing individuals and households who live below or above the poverty line when expenditure on health outstrips their income level.

##### Sustainability

OOPs depend on the ability and willingness to pay for services by the users. This, combined with the tendency of OOP to impoverish households, make it highly unsustainable.

#### Community-based health insurance (CBHI)

##### Efficiency

Majority of the respondents were of the opinion that CBHIs are inefficient. Also, one government official highlighted the reason for this to be linked to use of external technical support.

*“Most CBHI schemes are not meeting these criteria, and depend on technical facilitators like HMOs and not on community structures”.*

##### Equity

Respondents were not definitive on how equitable the scheme is.

##### Quality of service

It has been suggested that community structures at the LGA should be used to supervise service delivery and raise community voice to demand for quality.

##### Prevention of households from incurring catastrophic OOP

The schemes provide financial risk protection for the enrollees on the basic packages they offer, but may not provide cover at higher levels of care. As a government official clearly stated;

“*It has a weak structure and we need to be aware of the limits and know that CBHI is not a panacea for financial risk protection for the poor. In the long term, it is not going to work”.*

##### Sustainability

Based on the modality of their operations, sustainability of CBHIs is questionable because the pools are not large and they highly depend on subsidies. As a government official pointed out, among existing CBHIs in the country; “*Igboukwu CBHI is sustained by social solidarity and community effort while Kwara and Lagos CBHI schemes are sustained through government and donor subsidies”.*

It was suggested that CBHI is not a viable sole strategy for UHC because pools are small, fragmented, and are not sustainable without subsidies. Administrative cost is also not manageable except schemes are consolidated. In the words of a respondent,

*“It is better to start pooling at the LGA or State to ensure risk equalization. Rwanda consolidated their CBHI schemes, then evolved and moved to social security. No luxury of experimenting with pilots in a large and complex country like Nigeria”* (Development Partner).

### Analysis of what needs to change and future prospects for improved health financing in Nigeria

The opinions of the respondents are couched in the context of how the health financing environment can be modified so that the country adopts best practices in financial risk protection that could fast-track the attainment of universal health coverage. Table 4 highlights the needed changes in each of the major health financing mechanisms, and their perspectives on how these changes could occur given the commitments and contributions of key stakeholders.

**Table 4:** Needed change and future prospects of major health financing mechanisms in Nigeria

	What needs to change?	How can the change happen?
Government budget	Efficiency in health spending needs to be boosted. Wastages need to be reduced	• Use evidence to advocate for more budget for health. • Health spending should be results-driven and performance-based using relevant evidence (learn from private sector and donors)
	Need to improve transparency and strengthen accountability mechanisms	• Institutionalize Health Accounts to publicly disclose budget performance and make information accessible to citizens • CSOs to do more budget tracking and advocacy and promote citizens’ demand for government accountability
	Enhance sustainability	• Expand revenue streams (Tax administration and tax policies should focus on areas that need to be taxed e.g. informal and private sectors as has been done with success in Lagos state
OOP	Quality of care needs to improve, and users’ need to be unhindered in their choice of providers	• Establish systems that deliver quality and • promote consumerism (users should have a choice of where to purchase services without price discrimination)
NHIS	NHIS needs to be strengthened to perform its regulatory and quality assurance roles. HMOs need better regulation	• Capacity building of NHIS staff on implementation of regulatory frameworks. • NHIS should perform its oversight roles – enable them identify loopholes in the system and institute measures to block them
	Coverage of NHIS needs to improve and be expanded to the informal sector	• Amendment of the legislation that established the NHIS which made health insurance voluntary rather than mandatory. Engage States in the process. • CSOs should be engaged in sensitizing the informal sector
	Premium contribution by enrollees of FSSHIP	NHIS and government need to develop a proper advocacy tool to reach the end users (negotiations with labor unions)
	Lack of evidence of impact of NHIS	NHIS should commission a study to assess the program and produce evidence on impact
	Quality of care needs to improve	Revision of NHIS benefit package and essential drugs formulary to be more comprehensive
CBHI	Larger pools are needed to ensure risk equalization	Pooling should be done more centrally (e.g. at the LGA levels)
	Need for cross-subsidization to improve efficiency	Government should provide funds for subsidization. These could be earmarked for the poorest and most vulnerable groups
	Purchasing should be more strategic	Technical facilitators (such as NHIS, HMOs, trained MHAs) should be engaged to assist with purchasing
Donor funding	Public finance management needs to be strengthened for better coordination of donor funds	Review and revise the PFM system to enable donors bring their money into the system
	Sustainability of donor funds need to be improved through counterpart funding	State governments need to meet their responsibilities by paying counterpart funds
	Depend less on donor funding for delivery of certain health services	Explore other internal sources of funding for health care such as corporate social responsibility of private companies

## Discussion

The study found that health financing mechanisms are not operating optimally in Nigeria due to an interplay of political, economic, social, cultural and human factors. In addition, deployment of different financing mechanisms is not evidence-based or results-driven. Resources are not allocated equitably or in a manner that minimizes wastage and improves efficiency as was found in other studies [7]. Coverage of risk protection mechanisms is very low and preferentially benefits the rich. None of the mechanisms effectively protects individuals and households from catastrophic health expenditure.

In the case of government budget, its efficiency is affected by various factors, notably the very low budgetary allocation to health and disproportionately large share of allocation to personnel cost at the expense of actual services. Although evidence shows that epidemiological studies and prediction tools can help service planners allocate resources to support health programmes [27], health budget allocation is not based on epidemiological reports. Hence, quality of healthcare is suboptimal and services are neither patient-centred nor need-based.

With respect to the FSSHIP, coverage is very low [8, 12] and NHIS is not playing its regulatory and quality assurance roles effectively. Sustainability of FSSHIP is challenged by the fact that enrolees are currently not making their own share of contribution and the pool is fragmented. Attempts to integrate State government employees and harmonize schemes across States have met with resistance. There are also concerns that this could create or worsen inequities in resource allocation and access to quality services. Similar studies corroborate that NHIS has failed on many fronts to regulate the activities of HMOs within the FSSHIP [6, 12]. In the case of CBHI, there is inefficiency because activities are fragmented and pool sizes are too small and generally not viable without subsidies. This is worsened by their dependence on technical and social facilitators like HMOs for purchasing rather than on more cost-effective and sustainable community structures [19, 28].

The purchasing function of HMOs has been reported to be unsatisfactory and purchasing of health services has been mostly passive rather than strategic [6]. This is worsened by the fact that HMOs are not paid based on their productivity, rather on the number of enrolees allocated to them. Hence, there is no motivation to improve performance. The tendency for HMOs to capitalize on the fee-for-service design as a disincentive for referral from primary to secondary level care is another cause for concern when regulation is weak and HMOs are perceived to be more powerful than NHIS, the regulator.

Out of pocket payment was found to be the dominant health financing mechanism in Nigeria and this has also been reported by other studies [5, 6, 8, 12]. OOP is a very regressive way of purchasing healthcare, and of all other health financing mechanisms, it has the highest potential to lead to catastrophic health expenditure [5]. Regardless of this, there is no system to optimally harness and use OOP to deliver equitable and quality healthcare for citizens. People simply buy healthcare from wherever they find it and from whomever they can afford to buy from. Hence it does not promote consumerism or choice and needs to be completely replaced by better health financing mechanisms.

Donor funding has been a major source of financing for health service delivery particularly for vulnerable groups. It has also contributed considerably to financing of technical support and capacity building activities for health workers and health professionals. However, donor funding has not performed optimally because of weak coordination of donor activities by the Nigerian government. Sustainability of donor funding is also adversely affected by failure of some State governments to pay counterpart funds, or lateness in payment. The problem of donor fatigue is also affecting sustainability of donor-funded programmes. This calls for measures to address government dependence on donor funding through increased domestic resource mobilization for heath care financing.

Overall, this study highlights that for improved health financing in Nigeria, there is need for government to increase the health budget. Key decision makers should be involved in advocacy for more funding and for result- and evidence-driven financing. The needed changes in social health insurance include amendment of the legislation that established the NHIS which made health insurance voluntary rather than mandatory. Coverage should be expanded to include informal sector, and proper advocacy tools should be developed and used to negotiate with labor unions for government employees to start making their own contributions. Strategic purchasing should be the driver of service provision within the FSSHIP in order to improve its efficiency and equity [6]. Consumerism within the scheme should be improved by making beneficiaries aware of their rights. NHIS and FMOH should support State governments in establishing and managing State Health Insurance Schemes (SHIS) to minimize the apparent distrust between the State and Federal governments, and HMOs need to be properly regulated. With respect to government budget, efficiency can be improved by increasing budget allocation to health with focus on increasing domestic resources for health. Health spending should be more results-driven and output-based using relevant evidence. There is also a need for multi-sectoral collaboration in designing and implementing health financing strategies.

A major strength of this study is that information was obtained from national level stakeholders who have high level of influence in decision making for health financing. In addition, data were collected from actors who are most likely to use the findings in actual decision making. Furthermore, the qualitative approach enabled in-depth exploration of research questions. However, findings would have been enriched if more people at sub-national levels (including service users) were interviewed to better understand their experiences with implementation of different health financing mechanisms.

## Conclusions

All health financing mechanisms in Nigeria are performing at suboptimal levels. Resources are not equitably allocated or efficiently used to minimize wastage. Quality of services are perceived to be substandard, and individuals and households are not protected from catastrophic health expenditure. Given current situations of major health financing mechanisms in Nigeria in terms of health systems goals of efficiency, equity, quality of care and sustainability, financial risk protection for all citizens cannot be achieved. There are needed changes that must occur in order to improve health financing and ensure that Nigeria is on the right trajectory to achieving UHC. Key stakeholders in health financing, and decision makers that drive policy formulation and implementation need to make these changes in collaboration with non-health sectors.

## Additional file

**Additional file 1.** Topic guide for in-depth interviews.

## Data Availability

Datasets used and analysed during the current study are available from the corresponding author on reasonable request.

## Supplementary information

**Supplementary information** accompanies this paper at (10.1186/s12913-019-4512-4) contains supplementary material, which is available to authorized users.
