# Supplementary material for: Exploring effectiveness of different health financing mechanisms in Nigeria; what needs to change and how can it happen?
Source: BMC Health Serv Res. 2019 Sep 13;19:661. doi: 10.1186/s12913-019-4512-4 (PMC6743191; doi:10.1186/s12913-019-4512-4)
Supplement: Supplementary file 1 — Additional file 1. Topic guide for in-depth interviews. [file 12913_2019_4512_MOESM1_ESM.docx]

**In-depth interview topic guide**

| **One-on-one interview guide** |
| --- |
| Could you please describe to me in detail how health care is financed in Nigeria and/or in your state? In terms of:   1. Resource generation 2. Mobilization of funds 3. Pooling of funds 4. Purchasing of services |
| What health financing mechanisms could be feasibly implemented at the national and state levels in Nigeria? In what ways are they feasible? |
| Who is involved in health care financing in Nigeria and/or your state?  What roles do they play?  How do they interact with each other in performing their roles?  In what ways do these actors and their interactions with other actors  a. determine the level of funding that is available to the health sector in Nigeria  b. influence acceptability of different health financing mechanisms in different contexts  c. determine the effectiveness of health financing mechanisms to provide universal financial access to health services in Nigeria  Who should be involved in health care financing that is not? |
| What structure and institutional arrangements are in place for health care financing in Nigeria and/or your state? What roles do they play? How do they work together or interact in performing their health financing roles/functions?  In what ways do these structures/institutions and their interactions with other structures/institutions  a. determine the level of funding that is available to the health sector in Nigeria  b. influence acceptability of different health financing mechanisms in different contexts  c. determine the effectiveness of health financing mechanisms to provide universal financial access to health services in Nigeria  What structures ought to be in place that are not? |
| In your opinion, what are the major considerations in deciding on what health care financing mechanism to adopt in a place or for a population group? |
| What are policymakers and decision makers most concerned about when selecting health financing mechanisms? |
| What roles do politics and political factors play in these decisions? OR  What political interests drive actors’ decisions to accept and implement particular health financing mechanisms? |
| What roles do economic interests play in decisions to accept and implement particular health financing mechanisms? |
| What level of support can be expected for .... (Name the financial risk protection mechanism) in different contexts/localities/subpopulation groups in Nigeria and/or your state? |
| What factors in the Nigerian context hinder or could hinder .... (Name the financial risk protection mechanism)?  What factors in your state or localities/subpopulation groups hinder or could hinder ... (name the financial risk protection mechanism)?  How, if possible, could this be alleviated? And how would it affect achievement of universal financial risk protection in Nigeria and/or your state? |
